# Translational Potential into Health Care of Basic Genomic and Genetic Findings for Human Immunodeficiency Virus, *Chlamydia trachomatis*, and Human Papilloma Virus

**DOI:** 10.1155/2013/892106

**Published:** 2013-05-23

**Authors:** Jelena Malogajski, Ivan Brankovic, Stephan P. Verweij, Elena Ambrosino, Michiel A. van Agtmael, Angela Brand, Sander Ouburg, Servaas A. Morré

**Affiliations:** ^1^Institute for Public Health Genomics (IPHG), Department of Genetics and Cell Biology, Research School GROW School for Oncology & Developmental Biology, Faculty of Health, Medicine & Life Sciences, University of Maastricht, 6229 ER Maastricht, The Netherlands; ^2^Laboratory of Immunogenetics, Department of Medical Microbiology and Infection Control, VU University Medical Center, 1081 BT Amsterdam, The Netherlands; ^3^Department of Internal Medicine, VU University Medical Centre, 1081 HV Amsterdam, The Netherlands

## Abstract

Individual variations in susceptibility to an infection as well as in the clinical course of the infection can be explained by pathogen related factors, environmental factors, and host genetic differences. In this paper we review the state-of-the-art basic host genomic and genetic findings' translational potential of human immunodeficiency virus (HIV), *Chlamydia trachomatis* (CT), and Human Papilloma Virus (HPV) into applications in public health, especially in diagnosis, treatment, and prevention of complications of these infectious diseases. There is a significant amount of knowledge about genetic variants having a positive or negative influence on the course and outcome of HIV infection. In the field of *Chlamydia trachomatis*, genomic advances hold the promise of a more accurate subfertility prediction test based on single nucleotide polymorphisms (SNPs). In HPV research, recent developments in early diagnosis of infection-induced cervical cancer are based on methylation tests. Indeed, triage based on methylation markers might be a step forward in a more effective stratification of women at risk for cervical cancer. Our review found an imbalance between the number of host genetic variants with a role in modulating the immune response and the number of practical genomic applications developed thanks to this knowledge.

## 1. Introduction

Infectious diseases representa major health threat worldwide and a significant part of the burden of disease in developing countries [1]. Public health policy has traditionally had an important role in tackling such threat through established measures of prevention, mostly by controlling social and environmental determinants of health and through vaccination. With the recent advances in public health genomics, public health moved its focus from a “one size fits all” approach in health promotion and prevention activities to targeting populations and subpopulations with defined genetic risks and developed its unique role, translation of genome-based knowledge and technologies into public health policy and practice, and its integration across disciplines [2].

Scientific developments in basic research and the development of public health genomics have changed many paradigms regarding infectious diseases. Indeed, the recent evidence of genetic factors in the pathogenesis of infectious diseases transformed the view of such diseases from strictly pathogen-centric to the one incorporating host genetic determinants that modulate immune response. Though research in the field of genetic susceptibility to infectious diseases started in 1954, recent progress in genomics led to the characterization of molecular biomarkers and pathways as targets for diagnosis or intervention [3]. Furthermore, this understanding of infectious diseases explains the individual variation in susceptibility to an infection as well as the clinical course of the infection by pathogen related factors, environmental factors, and genetic differences. The field identifies genes responsible for influencing susceptibility to infections as well as their severity and response to treatment. This is predominantly achieved by studying candidate genes, genome wide associations, and twin studies [4].

A great amount of effort and resources have been directed to obtaining knowledge about host genetic components of infectious diseases and to confirm associations in order to develop genomic applications in everyday clinical practice and prevention. 

Nonetheless, although the amount of genetic data in relation to disease is increasing exponentially [5, 6], there is a clear lack of translation of such findings to healthcare applications. Indeed, the amount of information about basic genome-based scientific findings present in the scientific journals is disproportionate to the number of patents and marketed products used in hospitals [7].

In this paper, priority was given to three sexually transmitted diseases of significant public health relevance: HIV, HPV, and *Chlamydia trachomatis* (CT) genital tract infections.

The aim of this review is to provide a state-of-the-art overview on the translational potential of basic genomic and genetic findings related to HIV, CT, and HPV infections, into applications in public health focusing on their diagnostics and treatment.

## 2. Methods

Based on our field of expertise in sexually transmitted diseases (STDs) we selected the most prevalent bacterial STD *Chlamydia trachomatis* (CT) and the 2 most prevalent viral STDs *Human Papilloma Virus* (HPV) and the *Human Immunodeficiency Virus* (HIV) knowing that from these infectious diseases human genetic and genomic markers are described.

We used the HuGE Navigator (Version 2.0: an integrated, searchable knowledge base of genetic associations and human genome epidemiology (http://hugenavigator.net/)) [8] to identify papers with a description of potential translation on the basic findings of genetic and genomic markers into diagnostic applications and ultimately into public health. Identified papers and authors were expanded using PubMed searches. For each infectious disease a general introduction will be given, the key genetic and genomic markers will be described, and the translational potential outlined. Finally, a general discussion and conclusions will be provided. 

## 3. Results

### 3.1. HIV

Despite the decrease in incidence of HIV infection (in 2009 the number of newly infected individuals dropped by almost 20% compared to the previous year), the prevalence of HIV is still very high. At the end of 2009, it was estimated that there were 33.3 million people living with HIV. The growing prevalence and the reduction in the AIDS-related mortality are mainly attributed to the success of antiviral therapy [9]. Nonetheless, the public health relevance of the disease remains indisputable, as tackling HIV requires large financial expenditures, and it is still among the sexually transmitted diseases causing the highest morbidity and mortality and it is highly preventable [10].

As mentioned earlier, research in the field of infectious diseases has established that the susceptibility of an individual is also modulated by host genomic factors. In this context, recent genomic and genetic discoveries using candidate gene and genome wide association studies (GWAS) increased our knowledge of the association among genetic loci from the so-called “major susceptibility genes.” HIV infection is the most studied infection by the aforementioned approaches. The research of a genetic role for the individual differences in the course of infection, besides offering new strategies for developing a treatment or a vaccine, also provides basic insights in the immunopathology of the infection. Moreover, this newly collected evidence could provide an opportunity of identifying persons at higher risk of getting or progression of the infection. On the other hand, this could detect patients having genes that make them long-term nonprogressors, thus with delayed or no progression to AIDS.

#### 3.1.1. Review of the Host Genetic Variants Found to Influence HIV Infection

The review of papers written by the experts in the field of host genomic determinants of infection, disease progression, and disease outcome reveals the growing body of host genomic “suspects” by the year. However, few associations were positively confirmed. Among these, only 15–20% of observed genetic variants have been identified as influencing HIV infection [11].

Many studies and reviews place genetic variants of chemokine receptor and chemokine ligand genes, HLA and related genes on top of the list of influential genetic factors identified in HIV infection [11–16].

Chemokine receptors have an important role in modulating HIV-1 early infection. Particular attention has been given to *CCR5* and *CCR2* genes, encoding coreceptors on the surface of the CD4+ lymphocytes, crucial for HIV cell entry. In the initial stages of the infection, the HIV virus uses CCR5 as a preferred coreceptor [15]. As a result, a mutation in the chemokine receptor genes resulting in the absence or significant reduction of CCR5 molecules on the cell surface would have a protective effect. Indeed, the expression level of this coreceptor influences the HIV infection outcome, and mutation of this molecule is associated with the ability of the virus to enter the cells *in vitro*, the *in vivo* viral load, the CD4+ levels during highly active anti-retroviral therapy (HAART: combination of three or more antiviral drugs), and the progression of the diseases to AIDS. 

In 1996, it was discovered that the deletion of 32 base pairs of *CCR5* (*CCR5*Δ32) results in shortened and inactive proteins. So far, *CCR5*Δ32 remains the only discovered mutation that completely protects homozygotes from HIV infection and in heterozygotes slows down the progression of the disease [11]. Moreover, the discovery of *CCR5*Δ32 genetic variant opened the door for the development of a new type of anti-HIV medications. Data obtained from *CCR5* gene candidate studies have been rather timely applied in the pharmaceutical industry, leading to the development of novel therapies, as further discussed in the next section. 

In addition, the association between the +190 A>G mutation of *CCR2* chemokine receptor and the delayed onset of AIDS was discovered in 1997. The resulting substitution of the amino acid valine, at the position 64 of CCR2, to isoleucine influences HIV progression, but not the risk of HIV infection. HIV positive patients carrying this mutation showed delayed progression to AIDS by 2–4 years [17].

#### 3.1.2. Application of Research Based on Chemokine Receptors

As stressed earlier, the major goal of the research on host immunogenetics of HIV is to acquire knowledge of how differences in genetic variants are influencing individual susceptibility to infection and developing new drugs based on that. The research provided insights into the effects of CCR5 coreceptor blockade and downregulation on HIV infection [18]. As a result drugs with a new mechanism of action, the blockage of CCR5 receptors, were developed. These drugs are also known as entry inhibitors. So far there are only two approved such drugs in clinical use, Maraviroc (Pfizer) and Enfuvirtide (Roche) [19, 20]. Of the two, *Enfuvirtide* was the first to be FDA approved. The success of this drug, despite its proven antiviral efficacy in patients' treatment, was constrained by the difficulties related to its subcutaneous administration, causing skin abscesses. The first orally administered HIV entry inhibitor was *Maraviroc*, approved by the FDA for patients with R5 virus types in 2006. The drug binds to the CCR5 chemokine receptor causing a conformational change that blocks the gp41-mediated fusion of viral and cellular membranes [19]. The next most promising HIV entry inhibitor is *Vicriviroc* (Schering-Plough), a medicine with the same action mechanism as Maraviroc, but expected to be more effective. Vicriviroc has still not been approved by FDA, but phase III clinical trials have been recently completed [21].

A recent extensive review of HIV-1 entry inhibitors patented from 2004–2010, [20], revealed 35 small CCR5 antagonist molecules patented by 5 different pharmaceutical companies (Astra Zeneca, ViroChem Pharma, Anormed, Inc./Genzyme Corp., Euroscreen, and Ono Pharmaceuticals). In the same review, it was found that the number of patents for CXCR4 (coreceptors for X4 HIV strains) antagonists and dual CCR5/CXCR4 antagonists is significantly lower. Further, clinical developments of CXR4 antagonists have been delayed in preclinical and clinical studies due to serious side effects (cardiac abnormalities and liver toxicity) or lack of drug efficacy.

Human Leukocytes Antigen (HLA) genes encode proteins that present antigens to T and B lymphocytes. There are two classes of HLA genes: class I (loci A, B, and C) and class II genes. A strong association has been observed between HLA I alleles and protection/susceptibility to HIV [22]. The effect of HLA A, B, and C homozygosis in general is accelerated AIDS. Other confirmed associations include HLA alleles B*27 and B*57 and delayed progression to AIDS [15, 16, 22, 23]. On the other hand, the B*35 allele is associated with increased susceptibility and more rapid progression of the disease. The median time in which homozygous carriers of the B*35 allele develop AIDS is half the time of noncarriers of such alleles [24]. 

The association between genetic variants of HLA class I loci and CCR5 and the pathogenesis of HIV infection has been confirmed in recent years by many GWAS studies. However, GWAS did not identify further major susceptibility loci [25].

Association studies between HLA class II alleles and the susceptibility to the HIV infection has been less consistent. 

HLA genes have also been shown to have a role in the Mother to Child Transmission (MTCT) of HIV infection. Indeed, HLA class I concordance between mother and child is associated with higher risk of transmission, *vice versa* HLA discordance is associated with a lower risk [16]. 

#### 3.1.3. Application of Research on HLA Genes

Although none of the mentioned HLA genes have yet been identified as a target for new drugs, the information gathered on the disease progression modulated by different genotypes has provided valuable information for clinical trials [22]. Research on HLA alleles led to important pharmacogenetic applications. HLA B*5701 positive patients, who are at risk for hypersensitivity to *Abacavir* (a nucleoside reverse transcriptase inhibitor), cannot be treated with this drug. This serious, and possibly fatal, adverse drug reaction is present in 5% of patients [26]. Genetic testing of all the individuals before prescribing the drug prevents serious side effects, building a very strong case for a stratified medicine approach, tailored to individual genetic characteristics. The idea behind it is that our personal genetic differences create a need for accordingly different treatment approaches. In the case of Abacavir recognizing interpersonal variation in reaction to drug is an excellent example of stratifying HIV treatment based on genetic research. 

In summary, HIV immunogenetic research provided some basic insights into the immunopathology of the infection and gave foundations to the development of new drugs for the therapy of the infection. Ideally this will be just the first step in advancing therapies. Information on individual susceptibility, higher or lower individual risks, and delayed or accelerated AIDS progression associated with certain gene variants will make a more individually tailored treatment possible in the future.

#### 3.1.4. *Chlamydia trachomatis*

*Chlamydia trachomatis* is a leading cause for a variety of diseases including ocular, respiratory, and sexually transmitted diseases. This section of the review will only focus on the latter, since sexually transmitted *Chlamydia* infections are the most common worldwide, whereas, for instance, ocular infections are mostly seen in third world countries. Host genetic twin studies of *Chlamydia* have shown that 40% of the responses to *Chlamydia* are based on host genetics [27].

According to the WHO, “more cases of STD are caused by *Chlamydia trachomatis* than by any other bacterial pathogen” [28]. The persisting high incidence of 90–100 million cases per year worldwide makes *Chlamydia trachomatis* infection an enormous health problem throughout the world. The bacteria can be easily eliminated by antibiotic treatment; however, as a result of often being asymptomatic, the infection is frequently diagnosed too late or not at all. Infertility, premature delivery, PID, and ectopic pregnancy are some serious sequelae of the untreated infection [29].

Evaluation of the casual link between *Chlamydia* lower genital tract infection and tubal infertility is very challenging due to the fact that this is a “silent” complication, usually diagnosed years after the infection [30]. Infected women can either clear the bacteria without any damage to their reproductive functions or develop severe late complications, such as tubal occlusion and periadnexal adhesions, leading to infertility as the most severe of complications. The differences in disease outcome are often determined by genetic variations, such as single nucleotide polymorphisms (SNPs) in genes responsible for, amongst others, bacterial sensing receptors (and the pathways to which they belong) on cells such as macrophages as well as local vaginal and tubal epithelial cells. The higher the number of genes affected by SNPs, the more abnormal the immune response, leading to a higher chance of severe complications [31]. Inadequate recognition of the pathogen and consequent inadequate immune response lead to a higher risk of subfertility [32]. In a research performed on Gambian twins [27], it was estimated that 40% of variation in *Chlamydia* infection characteristics could be explained by differences in host genetic factors.

#### 3.1.5. Review of the Host Genetic Variants Found to Influence Chlamydia Lower Genital Tract Infection

*TLR Receptors.* Toll-like receptors (TLRs), with their role in identifying pathogens and initiating innate immune response, have been recognized as the most important factors in influencing differences in susceptibility to course and outcome of *Chlamydia* infection [33, 34]. Indeed, much of immunogenetic research in this field is focused on TLR genes and genes involved in their pathways, not only by mRNA- and protein-based studies but also by studying the association between SNPs in TLR genes leading to the loss of function of the receptors and the potential higher risk of late complications such as tubal infertility. The application of such research could be in the area of early diagnosis of tubal infertility or subfertility. Based on this evidence, the time now being lost as a result of late or misdiagnosis of tubal infertility could be directed to IVF attempts. 

So far, there are 10 TLRs identified in humans, recognizing different bacterial and viral components. TLRs activate signaling pathways of immune response against different pathogens by activating different inflammatory cytokines [35]. TLR2, TLR4, and TLR9 recognize pathogen-associated molecular patterns (PAMPs) of *Chlamydia trachomatis*. Genes for TLR receptors 2 and 4 are considered particularly important in modulating innate immune response to *Chlamydia trachomatis *[36]. 

Several studies showed that SNPs in *TLR4* have a role in making women more prone to subfertility as a late complication of *Chlamydia* infection. Nonetheless, the exact role of TLR4 in subfertility has not been yet clearly understood [33, 34]. Subfertile women who have IgG antibodies for *Chlamydia trachomatis* have a two times higher likelihood to be carriers of the *TLR4* +896 A allele, compared to women without tubal pathology [34]. Although this observation was not statistically significant, reported trends suggest that it could be worthwhile to further explore it in a larger cohort. Further, murine studies showed that TLR4 functional mice are more protected against reinfection compared with mice with dysfunctional or absent TLR4 [36]. In their study of genetic variants involved in the immune response regulation in genetic tract infections, Laisk et al. found that the *TLR4* +896 A>G and +1196 C>T polymorphisms protect against multiple infections with *C. trachomatis*, *N. gonorrhoeae*, *M. hominis*, *M. genitalium*, *U. parvum*, and *U. urealyticum*. Depending on the patient definition (i.e., including or excluding *C. trachomatis* serology), they found that specific *MBL2* high producing haplotypes can have a protection of a risk effect in tubal factor infertility. Low-producing *MBL2* haplotypes are associated with *C. trachomatis* serology positive tubal factor infertility patients [36].

In their study on the role of *TLR2* and *TLR4* in the development of tubal pathology on knock out (KO) mouse models, Darville et al. [33] showed that the amount of cytokines produced by macrophages depends on TLR2 but not on TLR4 receptors. Indeed, the deficiency of TLR2 receptors is associated with a decreased production of cytokines *in vitro*. *In vivo*, the deficiency or absence of TLR2 causes lower levels of inflammatory mediators, but the course of infection does not differ compared with naïve animals. Microscopic examination of the tubal tissue showed that mice with intact TLR2 are, however, more prone to the development of late inflammatory sequelae. Finally, their study concluded that TLR4 does not modulate innate immune response to *Chlamydia*, whereas *in vivo* experiments on TLR2 indicated its important role in protection against late inflammatory sequelae following *Chlamydia* genital tract infection [33]. 

In a study aiming at understanding the role of two *TLR2* SNPs in the susceptibility to infection and contribution to the development of the tubal pathology in Dutch women, Karimi et al. [37] revealed a statistically significant association between certain *TLR2* haplotypes and protection from tubal pathology and development of the late inflammatory complications (the absence of TLR2 is associated with an increase in the severity of the *Chlamydia* infection). 

*TLR9*—as already mentioned, most of the studies assessing host genetic determinants of *Chlamydia* infections are focusing on the extracellular TLR2's and TLR4's contribution to the differences in the susceptibility and severity of the infection. However, there is also an interest in the relevance of the intracellular TLR9. So far, human cohort data have not shown significant differences between carriers of mutant alleles and controls in the susceptibility to infection, course of the infection, or frequency of later tubal pathology. On the other hand, experiments in mice models found that *TLR9*-deficient mice had a higher level of protection against reinfection [38].

*HLA Alleles. *In addition to the research directed at *TLR* genes, there are also indications of association between tubal infertility caused by *Chlamydia trachomatis* and HLA alleles. Cohen et al. [39, 40] found that alleles of the HLA-DQ, DR1, and DRB5 loci modulate the severity of *Chlamydia* infections. Kinnunen et al. also found that specific HLA-DQ alleles are more frequently present in women with tubal infertility [41].

Besides the TLR and HLA alleles, in 2009, Morré et al. published an extensive overview of the then known genetic variants influencing susceptibility and severity of *Chlamydia* infections including SNPs in cytokines and other pathogen recognition receptors like NODs [42]. 

### 3.2. Application of Research

Immunogenetics research on *Chlamydia trachomatis* indicates that a proof of principle for the successful application of genetic and genomic markers for the prediction of late complications after the infection could have a strong public health impact.

Subfertility poses an enormous burden on healthcare and society throughout the world. Worldwide, 15% of couples trying to conceive suffer from subfertility [44, 45]. One of the major causes of female subfertility is tubal pathology (TP) [44], and CT is the single most common cause for infertility. If left untreated, CT may lead to ectopic pregnancy, tubal pathology, and ultimately infertility. The cost associated with subfertility is high, as it requires tubal surgery and *in vitro* fertilisation (IVF).

**Figure 1:**
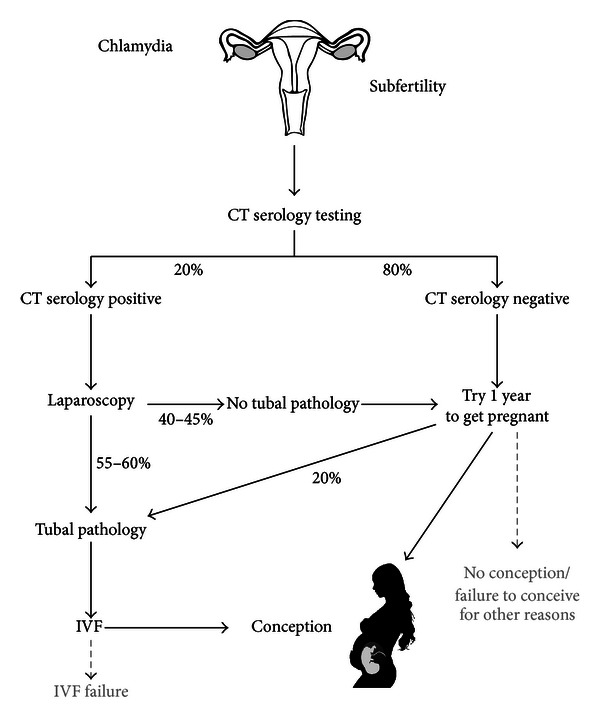
Current serology protocol for subfertility resulting from CT infection. Women with a negative CT serology are advised to try to conceive for one year; however, 20% of those women actually have tubal pathology and are thus misdiagnosed. Of the women with a positive CT serological test, 40–45% do not have tubal pathology after laparoscopic examination and are thus misdiagnosed. Figure adapted from Lal et al. [43].

Currently, CT IgG serology is used to assess the risk of CT-associated TP in subfertile women (20%) (Figure 1) [46]. CT serology has limited sensitivity and specificity and the predictive value is poor thus, many women undergo additional diagnostic procedures while not needed (40–45%) or do not get intervention while needed (19%). Laparoscopy is widely used to assess the risk of TP in women positive for CT IgG. This procedure is invasive and expensive (on average 3000 Euros including additional costs) and requires general anaesthesia. Furthermore, it holds a 1.5% risk of surgical complications (e.g., bleeding, infection, or worse).

Therefore it is crucial to develop a companion diagnostic to improve the assessment of risk of TP in CT-positive and negative women. By doing so, one is able to prevent invasive procedures in patients without TP and reduce both the cost and the psychological burden associated with laparoscopy. This companion diagnostic should merge serology, taking into account serological positivity and titres and considering new serological responses (e.g., pgp3) [47] and add the predictive value of host genetic markers involved, for example, related to the innate immune response to pathogens. The genetic trait should consist of a series of markers with a so-called SNP load or gene load linked to decision making for performing laparoscopy or not. Future studies should be directed at performing studies in larger cohorts to access the true clinical potential of this approach.

### 3.3. HPV

Roughly 20% of cancers are linked to various infectious agents [48]. Human papilloma virus (HPV) is one of these agents, and the role of different HPV subtypes in the etiology of cervical cancer has been well established [49]. HPV infections are in most cases cleared by the actions of the immune system within one year and often remain asymptomatic throughout that period. However, a small percentage of the infections eventually lead to some form of cancer. 

HPV-induced cancers account for approximately one-third of all cancers caused by infectious agents [50], and HPV is considered to be the most common sexually transmitted infectious agent [51]. However, studies have shown the existence of nonsexual modes of HPV transmission (including transplacental and transmission via fingers and objects [52–54]), and therefore, HPV cannot be referred strictly to as an STI [52]. 

The HPV virus infects skin or mucosal tissues in the anogenital area or the region of the head and neck. So far more than 100 types have been reported [50]. It has however been proven that approximately 15 out of these 100 types cause virtually all cases of cervical cancer [55]. Moreover, HPV types 16 and 18 account for around 70% of cervical cancer cases, and they—particularly type 16—have also been identified in anal, as well as some head and neck cancers [56]. 

The strong association between HPV infection and cervical carcinogenesis makes cervical cancer preventable, thus fulfilling an important criterion for public health relevancy. With the introduction of HPV vaccines, a major breakthrough in prevention has been made. Vaccines proved to be safe and efficacious [57] and vaccination programmes for girls and young women have been implemented in many countries. 

#### 3.3.1. Review of the Host Genetic Variants Found to Influence HPV Infection

Of all the women who are infected with HPV, only a small percentage develops cervical cancer. This observation suggests a role of host genetic factors influencing persistent HPV infections and progression into cervical cancer. 

*The Role of HLA*. Alleles have been reported to be associated with the development of HPV-related cervical cancer. In their review of evaluating this association, Hildesheim and Wang [58] found several alleles of HLA class II to be associated with an higher risk of developing cervical cancer (DQB1*03 alleles and DRB1*1501, DQB1*0602). As for HLA genetic variants' protective effect, several studies consistently reported that DRB1*13 and DOB1*0603 are associated with it [58]. Associations between HLA and HPV infection and progression to cancer are reported to be population- and HPV type-dependent. Indeed, HLA DQB1*0301 allele carries an increased risk of cervical cancer in the British population in case of infection with all HPV subtypes [59], while researchers in Bolivia found a statistically significant association of HLA DRB1*1602 with susceptibility toinfection [60].

In their recently published review of the genetic susceptibility to cervical cancer, Chen et al. [61] presented the most important genetic polymorphisms associated with the development of this disease. Their literature search identified, in addition to HLA genetic variants, genes encoding interleukin-1*β*, tumor necrosis factor *α*, interleukin-12 A and B, interferon-*γ*, interleukin-10, cytotoxic t-Lymphocyte antigen-4, p53, BRCA1, and LAMB3 as genes associated with persistent HPV infection and progression to cervical cancer [61]. In addition, certain genes encoding killer immunoglobulin-like receptors (KIR) also seem to be associated with cervical cancer [62]. 

So far, no genetic or genomic applications have been developed based on these findings. When it comes to applying genetic knowledge and discoveries into the field of HPV infection and cervical findings diagnosis and prevention, the strategy known as methylation takes the lead.

#### 3.3.2. The Role of Methylation

Methylation is a common mechanism through which the silencing of genes, and among these tumor-suppressor genes, can be achieved [64]. It represents a chemical alteration in regions of DNA referred to as “CpG islands,” commonly found in many promoter regions. The alteration leads to the inhibition of the transcription of genes controlled by such methylated promoters [65]. Methylation markers are easily detected in cervical scrapes, with, for example, methylation-specific PCR (MSP). Hence, positive MSP results in these samples are indicators of methylation of relevant genes in the tissue [65]. At the moment, the strategy for early detection of cervical neoplasia in screening programmes is cervical scraping cytomorphologic assessment (PAP test), which has a considerably low sensitivity. Data on sensitivity and specificity of the PAP test are highly heterogeneous. Depending on the study done and combination of tests and reference standard thresholds applied, they range from 18% to 98% for sensitivity and from 17% to 99% for specificity [66]. Furthermore, the National Cancer Institute assessed the sensitivity of the PAP smear to be 55–80% for high grade lesions and around 68% for low grade lesions [67]. Taking this into consideration, there is a need for the development of novel approaches, and additional tools based on methylation markers might be a step forward.

#### 3.3.3. Application of Methylation in Triage of Cervical Carcinomas

In the study by Henken et al. [65], 29 tumor-suppressor genes were analyzed as potential methylation targets, and 12 of them were found to have methylated gene promoters in cervical cancer tissue. Eight of those were also associated with consecutive stages in HPV-mediated transformation *in vitro*. The promoter that was most commonly methylated (in 92% of the examined carcinoma samples) was MGMT. 

**Figure 2:**
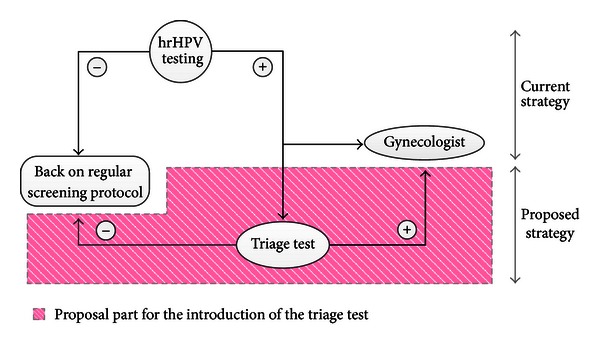
Introducing methylation as an addition to the primary hrHPV test would lower the number of unnecessary referrals to gynaecologists. Figure based on Yang et al. [63].

Methylation of the promoters CCNA1 and C13ORF18 in cervical scrapings is found to be strongly associated (*P* < 0.0005) with CIN2 (moderate cervical intraepithelial dysplasia) and higher grade stages of cervical dysplasia, as was determined in the study by Yang et al. [63]. Hence, these would be suitable markers for a triage test, referring a patient to a gynecologist upon a methylation-positive result. The more severe the lesion in the sample, the more methylation was present in these two gene promoters. Analysis of high methylation of these two markers has a high specificity (96% and 100%, resp.), as well as high positive predictive value. Further, Yang et al. [63] suggest that their methylation test should be used as a triage test in primary hrHPV testing (high risk HPV test identifies types of HPV which are linked to cervical cancer). hrHPV testing is more effective in preventing invasive cervical cancer; however, it is considered to be less sensitive than cytology in detecting CINs. Introducing methylation as a part of a triage test to the primary hrHPV test would lower the number of unnecessary referrals to gynecologists; especially in younger women who tend to be over diagnosed [68] (see Figure 2).

In another study evaluating the potential value of the methylation markers CADM1 and MAL as a triage tool for hrHPV+ women, it was found that there is a solid reasoning for combining markers which relate to different stages in cervical carcinogenesis [69]. They examined and confirmed the advantage of combining methylation patterns in the promoter region of more than one suppressor gene with the aim to increase the sensitivity for high grade CINs. A methylation-based test focuses on later phases of the carcinogenesis, given that these promoter alterations increase in these late stages. However, methylation-driven silencing of MAL promoter takes place at a very early point, before HPV-positive keratinocytes undergo tumor transformation. Whereas, silencing of CADM1 promoter by methylation correlates more with late stages. Overmeer et al. demonstrated that this marker combination is optimal for detection of CIN3 lesions [69].

In the process of progression into late stages, there are genes other than oncogenes and tumor suppressors also relevant. MicroRNAs (miRNAs) are short noncoding RNA molecules, which act in regulating expression of protein-coding genes, by pairing with sequences within such genes. hsa-miR-124 is an miRNA known to be silenced by methylation in many cancers, and Wilting et al. (2010) proved that this mode of silencing frequently occurs in cervical lesions as well [70]. No methylation was found in normal tissues, while almost 60% was detected in CIN3 lesions, and more than 93% methylation of hsa-miR-124 was present in cervical carcinomas. The methylation of this gene is not directly related to the presence of hrHPV. High positivity is however observed in CIN3 and cervical carcinomas, which altogether makes it a potentially very useful triage marker for hrHPV-positive women. This applies however not for setting where HPV genotyping is not implemented yet including under development countries.

Triage could serve as an additional step that would more aptly bridge screening and diagnosis in order for a better stratification of women at risk to be achieved [71]. It would be used on those with positive primary screening results to determine the further risk of the progression into later stages. 

The effects of constructing this type of triage test based on methylation would be expected to land a formidable impact on policies that currently regulate screening intervals.

## 4. Discussion and Conclusion

To our knowledge, this is the first review on the translational potential of basic genomic and genetic findings for HIV, CT, and HPV into applications in public health and in diagnostics, treatment, and prevention of late complications of these infectious diseases. We found scarce examples of the current application of genomic/genetic findings, in pharmacogenomics, and we found examples of genomic information with a promise of translation in the near future.

In our review, we did not focus on analytic validity, clinical validity, and clinical utility and other criteria generally considered to be the most important factors in evaluation of the genetic/genomic applications [72]. Since there are still no market-ready applications, so the aforementioned criteria could not be considered; we focused on an earlier step of this process. We focused on the promising examples of translation of the discovery into a possible application. 

Based on the review of the relevant literature some examples can be considered promising.

The genes responsible for susceptibility to HIV infection can be basically divided in two groups, chemokine receptors genes and HLA genes. So far, the discovery of the *CCR5*Δ32 genetic variant opened the door for the development of new anti-HIV drugs. Although undoubtedly a very important step forward, CCR5 targeted therapy and the research behind it are just one of the possible applications of immunogenetic information. Indeed, there is a significant amount of knowledge of certain genetic variants having a positive or negative influence on the course and outcome of HIV infection. Possible future use of the knowledge about the expected course of the infection would be advancing the standard of care and therapy after routine genetic testing.

In the field of *Chlamydia trachomatis* caused subfertility there is a promise for a more accurate subfertility diagnosis based on SNPs. Research showed that SNPs in *TLR4* possibly increase the risk of tubal pathology. Specific *TLR2* haplotypes are associated with protection from tubal pathology and development of the late inflammatory complications. 

These findings, together with the one carrying multiple SNPs in multiple pattern recognition receptors' (PRRs) encoding genes (*TLR9*, *TLR4*, *CD14*, and *CARD 15*/*NOD2*) doubles the risk of tubal pathology in *Chlamydia trachomatis* IgG-positive women compared to IgG-positive women carrying less than two SNPs, offer a proof of concept for the development of a genomic application in diagnosis of subfertility. 

A genetic test as a part of routine subfertility diagnosis should be able to save time and money by decreasing the number of unnecessary laparoscopies and the time patients unsuccessfully spend trying to get pregnant. 

In the field of HPV, there are some promising advancements in the early diagnosis of cervical cancer based on methylation tests. The methylation markers CADM1 and MAL were found to be an optimal combination for the detection of CIN3 lesions [69]. Moreover, the methylation of CCNA1 and C13ORF18 in cervical scrapings is found to be strongly associated with CIN2 and higher grade stages [63]. A triage test based on such methylation markers might be an important step towards a more effective stratification of patients at risk for cervical cancer.

The knowledge about the gene-disease associations should lead to growing numbers of genetic tests, which will in the future have an increasingly important role, in tailored clinical and drug treatment. However, in order for this translation process to succeed, the wide consensus among scientists, clinicians, policy makers, and the industry on necessity of going in this direction needs to be achieved [73].

Based on what we have shown here, there are many host genetic variants found to have a role in modulating the immune response to HIV, HPV, and *Chlamydia* infections. However, we found an imbalance between the number of host genetic variants with a role in modulating the immune response and the number of practical genomic applications. Thus, such new knowledge and technologies from basic research are not yet integrated in health in a timely, effective, and efficient manner [7]. 

This imbalance, the lack of translation from bench to bedside, is in favor of basic research that seems to be somewhat hermetic in quality, revealing confirmed positive association with a certain genetic variant and not exploring the future implications of these findings, should not represent a norm in the field. 

The next step is needed in which gene-disease association leads to the development of the genetic/genomic application. Starting with interdisciplinary collaboration is very important in the process of evaluation of role of genetic variants in the etiology of human diseases [74].

There are some clear and well-supported genetic associations with particular infectious diseases; these should be driving forces of the successful translation process.
